# The Interproximal Space and the Contact Point

**Published:** 1916-08

**Authors:** C. N. Johnson

**Affiliations:** Chicago, Ill.


### THE INTERPROXIMAL SPACE AND THE CONTACT POINT.

BY DR. C. N. JOHNSON, CHICAGO, ILL.

We look at the teeth in the mouths of our patients and frequently do not appreciate the significance of the things we are looking at. This is so particularly with the interproximal space, and especially with the contact point on the proximal surfaces of the teeth. This space has a peculiar form and an especial function. The form is in the shape of a pyramid with the base of that pyramid located at the alveolar process, and bounded laterally by the proximal surfaces of the teeth and the apex located at the proximal contact point. This space is normally filled with gum tissue. We see the teeth protruding from the gum, but we do not think of the significance of the large space down between those teeth.

The form of the gum tissue which fills this interproximal space performs an important function in the process of mastication. The gum tissue is in form of an arch bucco-lingually with the crest located at the contact point, and the slope of that arch is a wonderfully efficient provision in the process of the comminution of food. The food, when taken into the mouth, is divided by the teeth; part of it goes buccally and part lingually and is gathered up against the surfaces of the teeth by the tongue on one side and the cheek on the other, and we find a continuous process of cleansing by the friction of the food over the enamel. If you ever had occasion to extract a tooth, say a lower permanent molar on which upon the distal surface there was a perfect contact and a perfect interproximal space, and noticed after the extraction the form of the gum left between the first and second molars, you have seen that it comes up in a very thin edge with the top of the crest at the contact point.

Now, that space, on account of its important function, must be preserved in its normal form, or we shall not have a healthy gum tissue filling it.

### THE CONTACT POINT.

As to the form and function of the contact point. When we look into the mouth with the teeth arranged all in a row, we are apt to be misled as to the form and area of the contact point. It appears to the eye to be a broad contact, but the principle of the contact between the teeth should be a narrow, rounded contact, and as a matter of fact we have that in the normal teeth. The contact point between the bicuspids is manifestly small, but when it comes to the molars, as viewed in the mouth, this contact seems broader, on account of the greater bucco-lingual width of these teeth. But take two molar teeth out of the mouth and hold those teeth together the same as they would be normally together in the mouth and see how much light is obstructed between them, you will find it is the finest, smallest possible contact. As Dr. Black has described, it is about the area of two marbles held together. That is true with the molars, the same as the bicuspids. We get the impression of a broader contact point in the molars because they have a greater buccal width, but when you come to minutely examine the actual point of contact, it is in reality very small.

What is the general function of the contact point? We believe that the contact point is placed there for the purpose of guarding the interproximal space and the gum tissue in that space, and is there for the purpose of dividing the food and forcing it bucally and lingually. It is there for the purpose of protection of the gum in an entirely different way. You might say an individual might have teeth so tight together that food would never wedge between them. There is an individual movement in all teeth. It is impossible to build teeth so tightly together that at times fibres of food will not pass between the contact point. If we have a broad contact point, we will have those fibres held between the teeth; if we have a small, rounded contact point, they will not be held there, but will pass down into the interproximal space and the next excursion of food will wipe them right out and we will have that space kept clean, so that the maintenance of a proper interproximal space is absolutely dependent on a contact point of proper form.

I remember when this matter first began to be broached that one gentleman advocated that we should build a broad contact point in our fillings, but should build them so tightly together proximally or put a filling so tightly against the tooth next in line that fibres of food would not wedge between them. That is an utter impossibility. You get fibres of food coming between the teeth at times, and you will get them slipping past the contact point in spite of everything, on account of the individual movement of the teeth, one against the other, and in spite of this broad contact he mentioned, food will be retained there. And this gentleman later, when converted to the faith of a narrow contact point, was broadminded enough to acknowledge that he had been incorrect in his statement.

### EFFECT OF FAULTY INTERPROXIMAL SPACE AND CONTACT POINT.

Now, let us consider the deleterious effect of the passage of remnants of food between the contact point into the interproximal space.

We have, of course, to consider the danger of caries upon such proximal surfaces. Those surfaces, if kept clean, will not decay any more readily than the surfaces upon the occlusal third of a bicuspid, or a molar, unless there is a developmental defect. In these interproximal spaces we know we have many instances of caries because there is a little harbor or shelter there in which the microörganism of caries can work and form its acid. If we keep food out of the interproximal space, decay will not occur in that particular point. If we have lost the contact point, we will find a slight dropping in of the tip or crest of the gum tissue in the space, making an inverted arch, and a catch basin for food which is followed by decay in the proximal surfaces of the teeth due to this loss of proper contact.

We find a class of cases of caries the most distressing that I know of ensuing in some of these cases, presenting a decay, not beginning immediately rootwise of the contact point, but near the gingival region in the proximal surface. This frequently happens on the distal surface of the lower second molar following the loss of the first permanent molar. I want to impress upon every man and woman here the necessity of saving that first permanent molar in every case that you can. If that tooth be lost there follows inevitably a tipping forward of the second permanent molar, and you will find some time later that individual will come to you complaining of discomfort upon that side of the mouth. You look the teeth over and they may appear perfectly sound.

I had a case just recently which was typical of that condition, referred to me by a friend in the profession. The patient came to me and said. "My dentist has been trying to destroy the pulp in this tooth." The tooth appeared to be perfectly sound and still the patient complained of pain in that lower second permanent molar. The dentist in desperation started to destroy that pulp to give the man relief. He had not been able to get much more than through the surface of the enamel on account of the sensitiveness. When the patient came to me I ran the instrument down deeply on the distal surface of the tooth and found a large cavity never suspected, filled with gum tissue. Such cavities I find more and more frequently. If I can not see evidences of decay upon the exposed surfaces of a tooth which is uncomfortable,I probe under the gum. In the case mentioned I was able to give the patient relief. The reason we find such cavities is because the proper contact is lost between the teeth and because the interproximal space loses its correct form. Furthermore, there is another damage done, that is worse than a carious cavity in a tooth. You may find a cavity and fill it and save the tooth, or if crowns break off you can restore them, but if the surrounding structures of the teeth are lost the case is hopeless, and that is the chief significance of the maintenance of the interproximal space and contact point in normal conditions.

I have heard the statement made, and believe it is well within the limit, that eighty-five per cent. of the cases of pyorrhea alveolaris have been induced, in the first instance, by faulty proximal contact, and abnormal interproximal space; from food wedging in between the teeth that brings about, sooner or later—sometimes in a long time, sometimes very soon—a pyorrhea pocket, and you may have many cases of pyorrhea pockets beginning in the interproximal space, yet never recognize it.

We have been in the habit of looking over people's teeth, examining them for caries, and we think of nothing in the case but that little hole in the tooth. There are other things more important. We can control caries more easily than we can preserve the surrounding tissues of the teeth.

We must be more alert in the future as to this matter of food leaking in between the teeth. How many in this audience have ever experienced discomfort from fibres of food wedging in between the teeth? You are very modest about expressing yourselves, but I will wager that ninety per cent. of you have. Now, when you examine the teeth of a patient and find fibres of food in between the teeth you should call the patient's attention to it. We frequently tolerate that kind of trouble, not realizing its significance, and not knowing it can be remedied.

When you find that condition, you will find an unhealthy gum tissue in the interproximal space, particularly if it has existed for a long time. The gum is wonderfully tolerant; if it was not, you and I would not be long in practice. Where the food has been attacking the gum for any length of time, you will find this abnormal gum tissue, and that is the beginning of the pyorrhea pocket. The moment you have this contact point broken down the teeth begin dropping together, and you have an abnormal condition of the gum tissue started at once. What should be your remedy? In the first place, you should have a very vivid appreciation of the correct form of the contact point of the normal teeth that you may reproduce that in the filling which may be inserted. Unless you have vividly in your mind such proper form, you will not be very likely to reproduce it. You must have a picture in your mind's eye of exactly how that tooth looked before it was decayed and you must build to that. Not only that, but if the tooth was not a good form originally, you must make a good form of it. Nature sometimes makes mistakes, and the result is a bad form of the tooth as related to the arch, and in consequence of that abnormality we have a condition favorable to caries. We should, as I have said, have in our mind a picture of the proper form and contour of that tooth and with our gold foil or other material restore it exactly to that form. In using amalgam we are limited in our ability to restore normal contact. We all use this material, and it is a good material for filling certain cavities, but its greatest limitation is that it is not hard enough for a contact point that will stand up against the abutting enamel, without being worn flat. When you take into consideration the amount of abrasion you find in the natural enamel where the teeth wear against each other you can readily understand what may happen to an amalgam filling.

A gold foil filling is better, particularly if it is malleted very strongly at the contact point; but a gold inlay is the best form of restoration so far as the contact point is concerned, and I want to say of the man who introduced the gold inlay that he is a great benefactor to the profession in this matter of proper contact and the protection of the interproximal space.

First of all, you must secure your space by wedging. I am not going into the technical description of this procedure. I just wish to mention one thing, which probably you all use, and which I wish you would use oftener—the ordinary base plate gutta-percha for wedging the teeth apart. I would not use a bit of India rubber, which we used for years, but which I have discarded for the last fifteen years, because of its tendency to creep up into the interproximal space and injure the gum. If you wedge with India rubber, by all means place something over the gingival wall of the cavity in the way of gutta-percha or something of that kind to prevent the India rubber getting up in the interproximal space and impinging on the gum tissue. You must spring the teeth as far as you can by the separator without much discomfort to the patient, and then pack the gutta-percha in the cavity, making the gingival wall flat so the gutta-percha will not slide out into the space. Then build it tight against the proximal surface of the next tooth and build it sufficiently so there is an impingement of the opposing cusps of the teeth against the gutta-percha. Two or three days will be sufficient to separate most teeth beautifully. Sometimes I have left this gutta-percha under those conditions for a long time. If you protect the gum tissue and the interproximal space, you can leave that gutta-percha in there for a few months without doing any damage. It wedges slowly, it does not set up any irritation or soreness, and you can get a space between the teeth by wedging them in that way, without pain.

Be sure, before you begin your operation, that you have wedged the teeth far enough apart so that you can make a restoration that will give the gum tissue in the interproximal space proper protection. If you press the gum back in this way with gutta-percha and do not lacerate it in the operation the tissue will come back in place within twenty-four hours after the removal of the separating material and recover the gingival portion of the filling.

### VALUE OF THE GOLD INLAY IN RESTORING CONTACT POINT.

I am going to speak particularly of gold inlays, as I am devoted to the use of the gold inlay in restoring contact points, in molars and bicuspids.

Where the teeth have drifted together you have a loosened contact of the teeth on the opposite sides. The contact should be maintained in the entire arch as tight as you can get it. When you are called upon to insert an inlay or filling in a case where the contacts have loosened you should wedge the teeth till you tighten the contacts on either side and then you should make the mesio-distal width of the inlay a little bit greater than would seem necessary to make a tight contact, and force that inlay in every case until the patient complains. When you drive the inlay to place, the patient will say: "That is too tight, doctor; I can't stand that." But you can assure them that it will not be uncomfortable any appreciable length of time. In doing that you not only secure a good contact in the tooth you are operating upon, but tighten the contact of the other teeth on that side. I look upon the tightening of the contact all along the arch as one of the important things. In adult life we may have such a wearing on the proximal surfaces of the teeth, there is more and more opportunity for food to wedge between the teeth. This is a condition which no patient would tolerate any length of time if brought on suddenly, but as it comes on gradually we are apt to tolerate anything. It is our duty to discover that condition. If you find a patient with a condition of this kind resulting in pyorrhea pockets, the first thing you want to do is to correct the contact, before you begin to start your treatment of the pyorrhea. You will make no progress at all in these pyorrhea cases if you permit the food to wedge between the teeth upon the gum margins.

It is sometimes advisable to make the contact point on the gold inlay of 18 carat solder, flowed over the 22 carat inlay. It is necessary where we find these greatly abraded facets in the molars to make this contact point harder than the 22 carat gold will do; in fact, you can not make it any too hard. And the observance of this method of procedure will change a mouth that has been uncomfortable to one that is comfortable for mastication, and will give you a chance to cure any pyorrheal condition that may be present.

It is a very deceptive thing to look in the mouth and judge properly the proximal contact relations. The teeth are covered with saliva and you can not take the teeth out and look at them; but, you can test the contact relations of the teeth in the mouth with a silk ligature. We do not often enough apply any such tests.

If you find a place in the mouth where applying the test, the silk breaks, there is something wrong. It may be a deposit of salivary calculus or serumal calculus; it may be a faulty, and frequently is an imperfect form of filling; it may be a worn facet upon the proximal surface of the teeth, leaving an abrasion that is ragged. When you come to a condition of that kind it should be remedied, no matter what it is, because we must look more and more in the future to the protection of the gum tissue in that interproximal space if we are going to render adequate service to our patients.

In some cases where you have made good fillings, and in other cases of teeth that have never been decayed, where the contact point seems to be perfect, the individual is troubled with fibres of food wedging between the teeth. It is a rare thing, but it occasionally happens. I have had an experience in my own mouth and had my lesson from that, and I am going to give you my experience.

Between two lower bicuspids fibres of food began to lodge and I tested the contact point and it was perfect, and I was troubled for a time thinking that my theory was being proven unsound. Finally it occurred to me to take a bite in modeling compound and study the model, and when I came to study my models I found that the lingual cusp of the upper bicuspid had tilted a little, so that it came down between those two biscuspids in the form of a wedge, and whenever I closed my teeth upon fibrous food it would spring the lower bicuspids apart and force the food in. I ground off that lingual cusp of the upper bicuspid, making it flatter, and *presto!* I had no more fibres of food wedging into that space. You would think a man would learn something from that. I believe my patients were benefited by this experience, because in cases where I was unable to detect a faulty contact point and food was wedging I would look to the opposing tooth for the cause of the trouble, and ordinarily I would find a cusp that would spring the teeth apart, and by grinding that off I was able to give relief.

A few years later I again began to have trouble with food wedging in between the upper second and third molars in my mouth, and you would think that my former experience would at once suggest the trouble; but a man is an ignoramus when it comes to caring for himself. I figured that it was a bad form of contact. As I said a moment ago, nature sometimes makes a mistake in giving forms to teeth, and I believed she had made a mistake when she came to develop those molars. I thought I might have to go on digging fibres of food from between my teeth all my life, and, in fact, I did suffer from that condition for a year, until it occurred to me to take a bite, and I found that the lower molar had worn into a very effective wedge shape. I went to the operating room and took a stone, and was not as gentle grinding off that cusp of my own tooth as I would be with a patient, because I was so exasperated with myself. Some day some other such trouble will come at another place and will begin to bother me, and I am going to watch for it, and not going to suffer a year again before I will test the teeth in a model to see what is going on.

That is the plea I make to you today. Whenever you find this condition confronts you, particularly with this matter of discomfort from food wedging between the teeth, if you find there is a good contact point to begin with, if the teeth are normal, and the mesio-distal width of the interproximal space is normal and the gum tissue in it is normal, then you must look to the opposing tooth, and if you can not see by observation, make a model and then look at it from the lingual surface and you will frequently find there is some material tilting of the tooth, some little change, some wearing constituting a wedge on the part of that opposing tooth that is springing the other two teeth apart.—*Western Dental Journal.*

